# The cardiovascular risk factors associated with the plaque pattern on coronary computed tomographic angiography in subjects for health check-up

**DOI:** 10.1186/s40885-017-0062-4

**Published:** 2017-03-02

**Authors:** Dong-Hyeon Lee, Ho-Joong Youn, Hae-Ok Jung, Kiyuk Chang, Yun-Seok Choi, Jung Im Jung

**Affiliations:** 1 Division of Cardiology, Department of Internal Medicine, College of Medicine, Seoul St. Mary’s Hospital, The Catholic University of Korea, #505 Banpo-dong, Seocho-gu, Seoul, 137-701 Korea; 2 Radiology, Seoul St. Mary’s Hospital, The Catholic University of Korea, #505 Banpo-dong, Seocho-gu, Seoul, 137-701 Korea

**Keywords:** Computed tomography, Coronary artery disease, Atherosclerosis, Calcium, Dual-source CT

## Abstract

**Background:**

Although it is known that coronary computed tomographic angiography (CCTA) offers highly negative predictive value to exclude obstructive coronary lesions, the plaque pattern on CCTA has not been fully understood. The purpose of this study was to explore the difference of the plaque patterns on CCTA and to assess the cardiovascular risks in healthy subjects.

**Methods:**

A total of 3914 subjects (mean age: 55 ± 10 years, M : F = 2649 : 1265) who underwent CCTA for health check-up between January 2009 and December 2012 were enrolled. According to coronary artery calcium score (CACS) and plaque pattern on CCTA, subjects were categorized into four groups (group 1: normal; group 2: “non-calcified” plaque; group 3: “calcified” plaque; group 4: mixed plaque). We analyzed cardiovascular risks and Framingham risk score (FRS) among the groups.

**Results:**

The incidence of each group was group 1 in 55.0% (2152/3914), group 2 in 5.1% (200/3914), group 3 in 8.2% (319/3914), and group 4 in 7.2% (280/3914), respectively. There was no difference of FRS among the groups (6.4 ± 6.4%; 6.5 ± 4.6%; 8.2 ± 5.8%; 7.7 ± 5.7% *p* = 0.086). In multivariate analysis, HbA1c (OR = 2.285; 95%CI = 1.029 - 5.071; *p* = 0.042) in group 2; age (OR = 1.115; 95%CI = 1.034 - 1.202; *p* = 0.005) and smoking status (OR = 3.386; 95%CI = 1.124 - 10.202; *p* = 0.030) in group 3; and age (OR = 1.054; 95%CI = 1.011 - 1.099; *p* = 0.014) and hypertension (OR = 3.087; 95%CI = 1.536 - 6.202; *p* = 0.001) in group 4 were independent factors.

**Conclusions:**

Our data suggest that more individualized therapy for reduction of cardiovascular risks associated with plaque pattern on CCTA could be considered in healthy subjects.

## Background

Now it is well known that coronary artery disease (CAD) is the leading mortality cause due to sudden death or myocardial infarction in healthy subjects and a major public health problem in the world [1]. Therefore, it is potentially important that the endeavor to identify subclinical coronary atherosclerosis can lead to reduction in the rate of cardiovascular events. Although Framingham risk score (FRS), 10-year risk for coronary heart disease (CHD) represents a very useful diagnostic tool, there are some limitations for the estimation of the risk of cardiovascular morbidity and mortality such as overestimation in a low-risk population or underestimation the risk in a high-risk population [2].

Conventional invasive coronary angiography has been the standard method for diagnosing CAD [3, 4]. In addition, the recent multidetector coronary computed tomographic angiography (CCTA) using dual-source computed tomography (DSCT) was introduced as a useful, non-invasive tool for the evaluation of coronary atherosclerosis and the prediction of cardiovascular morbidity [5, 6]. However, the screening of coronary artery calcium score (CACS) using CCTA should not be recommended in asymptomatic individuals with low-risk (0 to 1 risk factor or a 10-year risk <10%) or high-risk (CHD risk equivalents or a 10-year risk >20%) according to Framingham criteria, but considered useful in patients with intermediate- risk (more than two risk factors or a 10-year risk 10-20%) [7, 8]. Nevertheless, CACS has not only an excellent negative predictive value to exclude the presence of significant CAD [9, 10], but provides also more important prognostic information for cardiovascular risk stratification than the biomarker, such as C-reactive protein [11, 12].

Until now, the usefulness of CCTA for estimating and predicting of subclinical coronary atherosclerosis in healthy subjects has not been well established, although past studies mostly included symptomatic patients with significant or obstructive CAD including acute coronary syndrome [13, 14]. The purpose of this study was to explore the difference of the plaque patterns on CCTA such as “non-calcified” or “calcified” plaque and to assess the cardiovascular risks in healthy subjects.

## Methods

### Study population

Between January 2009 and December 2012, a total of 3914 subjects (mean age: 55 ± 10 years, M : F = 2649 : 1265) who underwent CCTA for health check-up at the Heath Promotion Center of Seoul St. Mary’s Hospital (The Catholic University, Seoul, Korea), were enrolled.

**Fig. 1:**
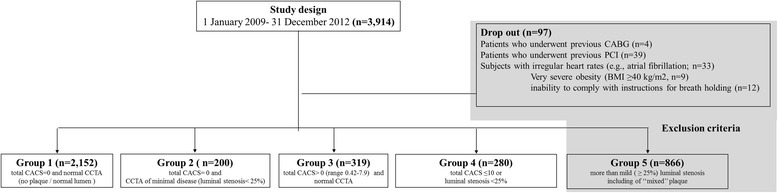
Flow diagram. Group 1 (e.g., normal lumen / no plaque) represented as “normal” coronary arteries: CACS = 0 & normal CCTA; Group 2 (e.g., <25% lumen / “non-calcified or soft” plaque): CACS = 0 & CCTA of minimal disease (luminal diameter <25%); Group 3 (e.g., <25% lumen or plaque with negligible impact on lumen / “calcified” plaque): CACS >0 & normal CCTA (CACS rage in our data: 0.42–7.9); Group 4 (e.g., <25% lumen / “mixed” plaque): CACS ≤ 10 & CCTA of minimal disease (luminal diameter <25%); Group 5 the subjects with CACS >10 or more than mild (≥25%) luminal stenosis [16–18]. CABG, coronary artery bypass graft; PCI, percutaneous coronary intervention; BMI, body mass index; CACS, coronary artery calcium score; CCTA, coronary computed tomographic angiography

Criteria for exclusion included: the patients (1) who underwent prior coronary artery bypass graft (CABG: *n* = 4, 0.10%); (2) who underwent prior percutaneous coronary intervention (PCI: *n* = 39, 1.0%) using stents, or the subjects (3) with irregular heartbeats (e.g., atrial fibrillation), very severe obesity (body mass index; BMI ≥40 kg/m^2^) [15], or inability to comply with instructions for breath holding; or (4) with CACS >10 or more than mild (≥25%) luminal stenosis represented as group 5, as a dropout (Fig. 1) [16–18].

According to the coronary artery calcium score (CACS) and plaque pattern on CCTA, we assigned as a inclusion criteria, subjects were categorized into four groups: (1) group 1 (e.g., normal lumen / no plaque) represented as “normal” coronary arteries: CACS = 0 & normal CCTA; (2) group 2 (e.g., <25% lumen / “non-calcified or soft” plaque): CACS = 0 & CCTA of minimal disease (luminal diameter <25%); (3) group 3 (e.g., <25% lumen or plaque with negligible impact on lumen / “calcified” plaque): CACS >0 & normal CCTA (CACS rage in our data: 0.42–7.9); (4) group 4 (e.g., <25% lumen / “mixed” plaque): CACS ≤ 10 & CCTA of minimal disease (luminal diameter <25%) who met the inclusion criteria, were included in the study [16–18]. After ruling out the subjects with CACS >10 or more than mild (≥25%) luminal stenosis represented as group 5 in present study, we compared FRS and the other traditional cardiovascular risks among the four groups.

This study was approved by the Institutional Review Committee of St Mary’s Hospital, the Catholic University of Korea and conducted in agreement with the Declaration of Helsinki. The participants were informed of the investigative nature of the study and written informed consent was obtained before enrollment (**XC11RIMI0091S**).

### Anthropometric parameters

Each participant also underwent a complete physical examination including anthropometric measurements. Heights were measured to the nearest 0.1 cm with a portable stadiometer (InBody 720; Biospace Ltd., Seoul, Korea) and body weights were measured to the nearest 0.1 kg using a digital scale wearing a standardized health check-up gown. BMI was calculated as the weight in kilograms, divided by the height in meters squared. Systolic blood pressure (BP), diastolic BP, and heart rate were measured using an automatic sphygmomanometer (BP203RV-II; Nippon Colin, Komaki, Japan) with subjects in a seated position after resting quietly for 10 min.

### Biochemical parameters

Blood samples were taken at health check-up day. The lipid profile, including total cholesterol, triglyceride, high-density lipoprotein cholesterol (HDL-cholesterol), and low-density lipoprotein cholesterol (LDL-cholesterol) levels were measured using enzymatic method by an automatic analyzer (7600-210; Hitachi Medical Corp., Tokyo, Japan). HbA1c was measured by G8 HbA1c analyzer (Tosoh Corporation, Tokyo, Japan). The biochemistry including fasting blood glucose and C-reactive protein from blood samples were measured by a biochemistry analyzer (7600-210; Hitachi Medical Corp., Tokyo, Japan).

### Framingham risk score

The 10-year risk for myocardial infarction and coronary death is estimated from total points and all participants were categorized into three different CHD risk groups according to the National Cholesterol Education Program (NCEP) guidelines: (1) low-risk (10-year risk <10%); (2) moderate- or intermediate- risk (10-year risk 10–20%); and (3) high-risk group (10-year risk >20%) [19].

### Coronary artery calcium score assessment

We measured coronary artery calcification with a DSCT (SOMATOM Definition; Siemens Healthcare, Forchheim, Germany). The participants did not receive additional premedications, such as β-blockers, for control of their heart rate. DSCT parameters were as follows: tube voltage = 120 kVp, gantry rotation time = 0.33 s, slice collimatio*n* = 64 × 0.6 mm, reconstruction slice width = 0.75 mm, reconstruction slice interval = 0.4 mm, kernel = B26f, field of view = 25 cm. Eighty mL of contrast agent (Iohexol, IOBRIX INJ 300; Tae Joon Pharm. Ind. Co., Ltd, Seoul, Korea) using a dual-head power injector (CT Stellant; Medrad Inc., Indianola, Pennsylvania, USA) was injected intravenously at 5 mL/s for 16 s.

All post-processing examinations were performed using retrospective electrocardiography (ECG)-gating. Scans were analyzed by consensus of two observers (**YS Choi** and **JI Jung**) with more than 3 years experience in cardiac CT imaging. CACS for vascular calcification were analyzed using a software (*syngo*.CT CaScoring; Siemens Healthcare; Forcheim, Germany).

For defining the quantity of coronary calcium, Agatston score, standard parameter, was used as the product of the area of calcification per coronary tomographic segment and a factor rated 1 through 4 dictated by the maximal calcium x-ray density in that segment, as described elsewhere [20]. The sum of all lesion scores for each major coronary artery including left main (LM), left anterior descending artery (LAD), left circumflex artery (LCX), and right coronary artery (RCA) was used to generate the total CACS.

### CCTA image acquisition and analysis

Reconstructed DSCT angiograms were analyzed on three- dimensional workstation (Advantage Windows Workstation 4.3, GE Healthcare, Milwaukee, Wisconsin, USA), using a software (Card IQ; GE Healthcare, Milwaukee. Wisconsin, USA). From a previously described standard American Heart Association (AHA) segmentation model [21], DSCT angiographic analysis was performed by **YS Choi** and **JI Jung** without knowledge of clinical findings. Two experienced intra- and inter- observers visually assessed each coronary segment using standard transaxial (2-dimensional) image stacks (“raw data”), oblique multiplanar reconstructions (MPRs), oblique maximum intensity projections (MIPs), curved multiplanar reformations (cMPRs), and volume-rendering (3-dimensional) technique (VRT) reconstructions [22], and performed manual computed tomography– based quantitative coronary analysis (CTQCA) using the most representative longitudinal image and a simplified calculation that estimates normal tapering of the coronary artery based on the initial method described by Reiber et al [23]. Maximal diameter stenosis severity was visually determined and were categorized as 0 to 5 (0 = no stenosis, 1 = 1% to 24%, 2 = 25% to 49%, 3 = 50% to 69%, 4 = 70% to 89%, 5 = 90% to 100%) [16–18].

### Statistical analysis

All data are expressed as mean ± standard deviation (SD) for continuous variables and as a frequency percentage for categorical variables and statistical analysis was performed using the SAS statistical software version 9.2 (SAS Institute, Cary, NC, USA). Analysis among the groups for continuous variables was performed using ANOVA test and analysis of categorical data was performed using the Tukey’s *b*-test as a post-hoc t-test. Analysis between the two groups was performed using unpaired *t*-test for continuous variables and *chi*-squared test for categorical data. The clinical variables related to FRS were assessed using Pearson correlation coefficient. To identify independent factors associated with the plaque pattern on CCTA, we used multiple logistic regression analysis and calculated odds ratios (OR) and 95% confidence intervals (95% CI). All statistical tests were 2-tailed and *p* <0.05 was considered statistically significant.

## Results

### Clinical characteristics

The mean age of a total of 3914 participants was 55 ± 10 years; there were more male (*n* = 2649) than female (*n* = 1265) subjects. A total of 977 of 1265 (78.8%) female subjects were postmenopausal. The prevalence of hypertension, diabetes mellitus, dyslipidemia, familial history of cardiovascular risk and current smoking state were 32.5, 12.8, 16.0, 84.2 and 20.3%, respectively.

Mean total CACS was 52 ± 200 mm^3^. Average scan heart rate was 67 ± 10 beats per minute.

Of 2951/3914 (75.4%) subjects enrolled for this study, the prevalence of each group according to the CACS and plaque pattern on CCTA was group 1 in 55.0% (2152/3914), group 2 in 5.1% (200/3914), group 3 in 8.2% (319/3914), and group 4 in 7.2% (280/3914), respectively. Baseline clinical characteristics including demographic data, laboratory findings and FRS in four groups are presented in Table 1.

**Table 1:** Baseline clinical and laboratory characteristics

Total = 2951	Group 1 (*n* = 2152)	Group 2 (*n* = 200)	Group 3 (*n* = 319)	Group 4 (*n* = 280)	*p* value
Demographic data					
Age, year *‡∥	52 ± 10	56 ± 9	57 ± 9	56 ± 9	<0.001
Gender, male, n (%) ¶	887 (41.2)	98 (49.0)	151 (47.3)	199 (71.1)	0.003
Hypertension, n (%) ¶	567 (26.3)	57 (28.5)	18 (5.6)	142 (50.7)	0.949
Diabetes mellitus, n (%)	191 (8.9)	30 (15.0)	9 (2.8)	27 (9.6)	0.359
Dyslipidemia, n (%)	284 (13.2)	26 (13.0)	15 (4.7)	44 (15.7)	0.569
Familial history of CVA, n (%)	573 (26.6)	61 (30.5)	24 (7.5)	93 (33.2)	0.245
Body mass index, Kg/m^2^ ‡∥	24.6 ± 3.7	24.9 ± 3.4	25.8 ± 4.1	25.6 ± 4.0	< 0.001
Systolic BP, mm Hg ‡∥	122 ± 14	127 ± 14	127 ± 17	126 ± 13	< 0.001
Diastolic BP, mm Hg *‡∥	72 ± 10	75 ± 10	76 ± 11	75 ± 10	< 0.001
Heart rate, beats per minute *	64 ± 10	65 ± 11	65 ± 10	64 ± 11	0.054
Smoking status, n (%)	94 (4.4)	6 (3.0)	13 (0.9)	78 (27.9)	0.003
Laboratory findings					
Total cholesterol, mg/dL	204 ± 36	198 ± 39	203 ± 40	204 ± 35	0.416
Triglyceride, mg/dL∥	116 ± 81	126 ± 99	120 ± 79	133 ± 79	0.045
HDL-C, mg/dL ‡∥	52 ± 13	51 ± 11	50 ± 12	49 ± 11	0.006
LDL-C, mg/dL *	125 ± 32	129 ± 30	125 ± 36	124 ± 33	0.313
Fasting blood glucose, mg/dL *‡	94 ± 21	102 ± 34	101 ± 24	99 ± 21	0.008
HbA1c,% *‡	5.6 ± 0.7	6.0 ± 1.4	5.9 ± 0.8	5.7 ± 0.6	< 0.001
C reactive protein, mg/dL	0.16 ± 0.41	0.16 ± 0.27	0.19 ± 0.32	0.18 ± 0.30	0.521

Data are expressed as means ± standard deviation or number of patients (percentage)

Group 1, CACS = 0 & normal CCTA; Group 2, CACS = 0 & CCTA of minimal disease (luminal diameter < 25%); Group 3, CACS >0 & normal CCTA (CACS rage: 0.42–7.9); Group 4, CACS ≤ 10 or CCTA of minimal disease (luminal diameter < 25%) [16–18].

* *p* < 0.05, *p* values was analyzed using Student’s *t*-test for the relationship between group 1 and 2

† *p* < 0.05, *p* value was analyzed using *chi*-square test for the relationship between group 1 and 2

‡ *p* < 0.05, *p* value was analyzed using Student’s *t*-test for the relationship between group 1 and 3

§ *p* <0.05, *p* value was analyzed using *chi*-square test for the relationship between group 1 and 3

∥ *p* < 0.05, *p* value was analyzed using Student’s *t*-test for the relationship between group 1 and 4

¶ *p* <0.05, *p* value was analyzed using *chi*-square test for the relationship between group 1 and 4

*CVA* cerebrovascular accidents, *BP* blood pressure, *HDL-cholesterol* high density lipoprotein cholesterol, *LDL-cholesterol* low density lipoprotein cholesterol

### “Non-calcified” plaque on CCTA

**Fig. 2:**
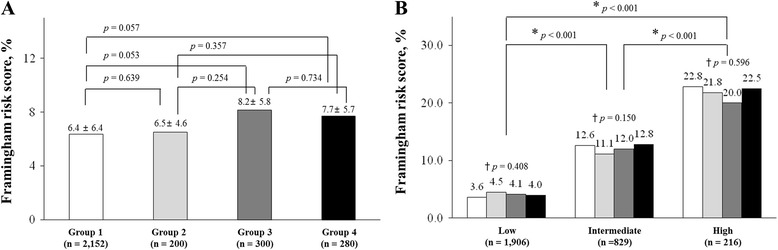
Comparison of Framingham risk score among the groups (**a**) and according to the risk stratification of Framingham risk score (**b**). Group 1, CACS = 0 & normal CCTA; Group 2, CACS = 0 & CCTA of minimal disease (luminal diameter <25%); Group 3, CACS >0 & normal CCTA (CACS rage: 0.42–7.9); Group 4, CACS ≤ 10 or CCTA of minimal disease (luminal diameter <25%) [16–18]. *, *p* value was analyzed using Student’s t-test for the relationship between two groups; †, *p* value was analyzed using ANOVA test for the relationship among the four groups. CACS, coronary artery calcium score; CCTA, coronary computed tomographic angiography

Age (56 ± 9 year vs. 52 ± 10 year, *p* <0.001), diastolic BP (75 ± 10 mmHg vs. 72 ± 10 mmHg, *p* <0.001) in demographic data, and plasma LDL-cholesterol (129 ± 30 mg/dL vs. 125 ± 32 mg/dL, *p* = 0.031), fasting blood glucose (102 ± 34 mg/dL vs. 94 ± 21 mg/dL, *p* <0.001) and HbA1c (6.0 ± 1.4% vs. 5.6 ± 0.7%, *p* <0.001) concentration in laboratory findings showed a significant difference between group 2 and 1, respectively (Table 1). However, there were no significant gender difference (group 1, M : F = 887: 1265; group 2, M : F = 98 : 102; *p* = 0.067) and in FRS (6.4 ± 6.4% vs. 6.5 ± 4.6%, *p* = 0.869; Fig. 2a) between group 2 and 1.

### “Calcified” plaque on CCTA

Age (57 ± 9 year vs. 52 ± 10 year, *p* <0.001), BMI (25.8 ± 4.1 kg/m^2^ vs. 24.6 ± 3.7 kg/m^2^, *p* <0.001), systolic BP (127 ± 17 mmHg vs. 122 ± 14 mmHg, *p* <0.001), and diastolic BP (76 ± 11 mmHg vs. 72 ± 10 mmHg, *p* <0.001) in demographic data, and plasma fasting blood glucose (101 ± 24 mg/dL vs. 94 ± 21 mg/dL, *p* = 0.001) and HbA1c (5.9 ± 0.8% vs. 5.6 ± 0.7%, *p* <0.001) concentration in laboratory findings showed a significant difference between group 3 and 1, respectively (Table 1). Plasma HDL-cholesterol (50 ± 12 mg/dL vs. 52 ± 13 mg/dL, *p* = 0.016) showed a significant lower in group 3 greater than group 1 (Table 1). However, there was no a significant gender difference (group 1, M : F = 887 : 1265; group 3, M : F = 151 : 168; *p* = 0.062) and in FRS (6.4 ± 6.4% vs. 8.2 ± 5.8%, *p* = 0.105; Fig. 2a) between group 1 and 3, respectively.

### “Mixed” plaque on CCTA

Age (56 ± 9 year vs. 52 ± 10 year, *p* <0.001), male ratio (71.1% vs. 41.2%; *p* = 0.012), prevalence of hypertension (71.1% vs. 26.3%; *p* = 0.038), BMI (25.6 ± 4.0 kg/m^2^ vs. 24.6 ± 3.7 kg/m^2^, *p* = 0.001), systolic BP (126 ± 13 mmHg vs. 122 ± 14 mmHg, *p* = 0.001), and diastolic BP (75 ± 10 mmHg vs. 72 ± 10 mmHg, *p* <0.001) in demographic data, and triglyceride (133 ± 79 mg/dL vs. 116 ± 81 mg/dL, *p* = 0.017) concentration in laboratory findings showed a significant higher in group 4 greater than group 1, respectively (Table 1). Plasma HDL-cholesterol (49 ± 11 mg/dL vs. 52 ± 13 mg/dL, *p* = 0.017) showed a significant lower in group 4 greater than group 1 (Table 1). However, there was no a significant difference in FRS (6.4 ± 6.4% vs. 7.7 ± 5.7%, *p* = 0.057; Fig. 2a) between group 1 and 4.

### FRS according to the plaque pattern on CCTA

There was no difference of FRS among the groups (6.4 ± 6.4% vs. 6.5 ± 4.6% vs. 8.2 ± 5.8% vs. 7.7 ± 5.7, *p* = 0.086; Fig. 2a). From the analysis for FRS using unpaired *t*-test, there was no difference in FRS [(group 1 : 2 = 6.4 ± 6.4%: 6.5 ± 4.6%, *p* = 0.639) vs. (group 1 : 3 = 6.4 ± 6.4%: 8.2 ± 5.8%, *p* = 0.053) vs. (group 1 : 4 = 6.4 ± 6.4%: 7.7 ± 5.7%, *p* = 0.057), respectively; Fig. 2a].

In group 1, age (*r* = 0.433, *p* <0.001), BMI (*r* = 0.221, *p* <0.001), systolic BP (*r* = 0.265, *p* <0.001), and diastolic BP (*r* = 0.320, *p* <0.001) in demographic data and plasma total cholesterol (*r* = 0.292, *p* <0.001), triglyceride (*r* = 0.324, *p* <0.001), HDL-cholesterol (*r* = -0.314, *p* <0.001), LDL-cholesterol (*r* = 0.315, *p* <0.001), and fasting blood glucose (*r* = 0.138, *p* = 0.017) in laboratory findings was closely related FRS, respectively (Table 2). In group 2, there was no cardiovascular risk factor related FRS (Table 2). In group 3, age (*r* = 0.562, *p* <0.001) in demographic data and plasma total cholesterol (*r* = 0.384, *p* = 0.030), triglyceride (*r* = 0.420, *p* = 0.017) in laboratory findings was closely related FRS, respectively (Table 2).

**Table 2:** Correlation coefficients between Framingham risk score and clinical variables

Total = 2951	Group 1 (*n* = 2152)		Group 2 (*n* = 200)		Group 3 (*n* = 319)		Group 4 (*n* = 280)	
	*r*	*p* value	*r*	*p* value	*r*	*p* value	*r*	*p* value
Demographic data								
Age, year	0.333	< 0.001	0.124	0.573	0.562	0.002	0.112	0.449
Body mass index, Kg/m^2^	0.221	< 0.001	0.261	0.228	0.056	0.764	0.124	0.401
Systolic BP, mm Hg	0.265	< 0.001	0.079	0.721	0.278	0.123	0.264	0.070
Diastolic BP, mm Hg	0.320	< 0.001	0.022	0.922	0.290	0.108	0.331	0.022
Heart rate, beats per minute	0.027	0.637	0.027	0.312	0.035	0.848	0.324	0.025
Laboratory findings								
Total cholesterol, mg/dL	0.292	< 0.001	0.050	0.822	0.384	0.030	0.110	0.456
Triglyceride, mg/dL	0.324	< 0.001	0.025	0.911	0.420	0.017	0.301	0.037
HDL-C, mg/dL	- 0.314	< 0.001	- 0.228	0.295	- 0.175	0.339	−0.356	0.013
LDL-C, mg/dL	0.315	< 0.001	0.053	0.809	0.337	0.059	0.116	0.434
Fasting blood glucose, mg/dL	0.138	0.017	0.005	0.980	- 0.076	0.678	0.228	0.119
Hemoglobin_A1c_,%	0.092	0.115	0.115	0.612	0.048	0.796	0.165	0.269
C reactive protein, mg/dL	- 0.015	0.802	0.116	0.628	0.270	0.212	0.166	0.277

Group 1, CACS = 0 & normal CCTA; Group 2, CACS = 0 & CCTA of minimal disease (luminal diameter <25%); Group 3, CACS >0 & normal CCTA (CACS rage: 0.42–7.9); Group 4, CACS ≤10 or CCTA of minimal disease (luminal diameter <25%) [16–18].

*BP* blood pressure, *HDL-cholesterol* high density lipoprotein cholesterol, *LDL-cholesterol* low density lipoprotein cholesterol

According to the NCEP guidelines, the prevalence of each subgroup for 10-year CHD risk was low-risk in 48.7% (1906/3914), intermediate-risk in 21.2% (829/3914), and high-risk group 5.5% (216/3914), respectively (Fig. 2b) However, there was no difference in FRS of the subgroup, including low-risk (group 1: 2: 3: 4 = 3.6%: 4.5%: 4.1%: 4.0%, *p* = 0.408), intermediate-risk (group 1: 2: 3: 4 = 12.6%: 11.1%: 12.0%: 12.8%, *p* = 0.150) and high-risk subgroup (group 1: 2: 3: 4 = 22.8%: 21.8%: 20.0%: 22.5%, *p* = 0.596), respectively (Fig. 2b).

### Independent cardiovascular risks

In multivariate logistic regression analysis for the cardiovascular risks, HbA1c (OR = 2.285; 95% CI = 1.029–5.071; *p* = 0.042) was an independent factor associated with group 2 (*so called* “non-calcified or soft” plaque); age (OR = 1.115; 95% CI = 1.034–1.202; *p* = 0.005) and smoking status (OR = 3.386 ; 95% CI = 1.124–10.202; *p* = 0.030) were independent factors associated with group 3 (*so called* “calcified” plaque); and age (OR = 1.054; 95% CI = 1.011–1.099; *p* = 0.014) and presence of hypertension (OR = 3.087; 95% CI = 1.536–6.202; *p* = 0.001) were independent factors associated with group 4 (*so called* “mixed” plaque), respectively (Table 3).

**Table 3:** Multivariate analysis

	Oddi ratio	95% confidence interval	*p* value
Independent factor associated with group 2, so called “non-calcified or soft” plaque			
Age, year	1.019	0.968–1.073	0.473
Gender, male	2.452	0.686–8.767	0.168
Systolic BP	0.977	0.918–1.039	0.455
Diastolic BP	1.052	0.973–1.137	0.201
LDL-cholesterol	1.009	0.995–1.005	0.218
Fasting blood glucose	0.980	0.947–1.015	0.260
HbA1c	2.285	1.029–5.071	0.042
Smoking status	0.988	0.352–2.773	0.981
Independent factor associated with group 3, so called “calcified” plaque			
Age, year	1.115	1.034–1.202	0.005
Gender, male	6.066	0.974–37.799	0.053
Body mass index	0.905	0.708–1.156	0.425
Systolic BP	1.039	0.985–1.095	0.159
Diastolic BP	0.982	0.919–1.049	0.593
HDL-cholesterol	1.011	0.979–1.045	0.500
Fasting blood glucose	0.994	0.970–1.018	0.593
HbA1c	1.681	0.817–3.459	0.159
Smoking status	3.386	1.124–10.202	0.030
Independent factor associated with group 4, so called “mixed” plaque			
Age, year	1.054	1.011–1.099	0.014
Gender, male	1.294	0.520–3.220	0.580
Hypertension	3.087	1.536–6.202	0.001
Body mass index	0.866	0.710–1.098	0.183
Systolic BP	1.002	0.960–1.072	0.954
Diastolic BP	1.014	0.969–1.005	0.410
Heart rate	1.027	0.993–1.061	0.119
Triglyceride	1.000	0.995–1.005	0.973
HDL-cholesterol	0.999	0.999–1.030	0.942
Fasting blood glucose	0.989	0.996–1.013	0.378
HbA1c	1.349	0.662–2.746	0.410
Smoking status	1.817	0.736–4.485	0.195

Group 2, CACS = 0 & CCTA of minimal disease (luminal diameter <25%); Group 3, CACS >0 & normal CCTA (CACS rage: 0.42–7.9); Group 4, CACS ≤10 or CCTA of minimal disease (luminal diameter <25%) [16–18].

*BP* blood pressure, *LDL-cholesterol* low density lipoprotein cholesterol, *HDL-cholesterol* high density lipoprotein cholesterol

## Discussion

The accumulation of atherosclerotic plaque without significant coronary stenosis happens over many years prior to acute cardiovascular events, including myocardial infarction or sudden cardiac death. Furthermore, CACS on CCTA, as a recent diagnostic tool, has been shown to be helpful in patients with low- and intermediate-risk who presents with atypical cardiac symptoms [7, 8]. At the same time, effective strategies for earlier identification of subclinical coronary atherosclerosis requires in healthy subjects. In the present study, unlike previous research using CCTA in symptomatic patients with significant or obstructive CAD [13, 14], we focused on the different cardiovascular risk factors associated with the plaque pattern on CCTA in healthy subjects. As a result, in this single-center, cross-sectional study of healthy subjects comparing CCTA with FRS, our data revealed that CCTA is reliable and effective for the estimation of the different cardiovascular risk factors associated with the plaque pattern on CCTA in healthy subjects. However, to predict the presence of subclinical coronary atherosclerosis, further investigations are required prospective study in larger populations via multicenter trials.

### Association with FRS and the plaque pattern on CCTA

FRS or CACS on CCTA for cardiovascular risk stratification is a useful tool. However, these tools alone may insufficient to identify subclinical coronary atherosclerosis in some part of the population. In addition, the combination of FRS and CACS may provide more accurate estimation of the risk of cardiovascular events [24]. In our study, although our data showed a weak correlation between FRS and traditional cardiovascular risk factors such as older age, obesity indicators, blood pressure and plasma lipid profile in subjects with “normal” coronary arteries on CCTA, there was no difference of FRS among the groups classified according to the plaque pattern on CCTA. As described in several previous published studies [7, 8], these findings are particularly consistent with the facts that CACS on CCTA in clinical application can provide valuable prognostic evaluation and serve as an important tool for cardiovascular risk stratification of asymptomatic or healthy individuals, although CACS on CCTA should not be recommended as a tool to diagnose significant obstructive CAD in symptomatic patients.

### Difference of the plaque pattern on CCTA

In analysis for the cardiovascular risk stratification of the development and progression of subclinical coronary atherosclerosis and the difference of the plaque pattern on CCTA such as “non-calcified” or “calcified” plaque, North et al. demonstrated the role of smoking status in the pathogenesis of “calcified” coronary plaque, similar to our result [25]. On the other hand, from the ROMICAT trial as a prospective, observational cohort study, Lehman et al. reported that smoking were independently associated with coronary atherosclerotic plaque burden progression on CCTA in patients with acute chest pain over 2 years, although rate of progression is dependent on plaque composition and may be higher for “non-calcified” when compared to “calcified” plaque [26]. In addition, in patients referred to the emergency department with chest pain, Yoon et al. reported that the patients ≥50% CAD of “non-calcified” plaque on CCTA were younger and had a higher prevalence of smoking [27].

Otherwise, the present study may have an important or interesting clinical implication in the association between HbA1c, as a key marker of diabetes control and “non-calcified” plaque from this observation. Interestingly, Hausleiter et al. demonstrated the role of “non-calcified” plaque, characterized by significantly higher total cholesterol, LDL-C, and C-reactive protein levels in patients with acute coronary syndrome [28]. Furthermore, more recent studies suggested that effective prevention has to be focused on the type of plaque composition [29–31]. Nicholls et al. demonstrated that “calcified” plaques are more resistant to undergoing changes in size in response to systemic interventions targeting atherosclerotic risk factors. On the contrary, “non-calcified” plaque might have a higher tendency to regress in response to established medical therapies [29]. Several studies also suggested that “mixed” plaque could convey a higher coronary risk including of acute coronary syndromes [29, 30]. Thus, to overcome these various issues and problems for identifying subclinical coronary atherosclerosis, we need to conduct further research in a larger population including of ethnic differences.

There are several limitations that our study includes the relatively small sample size and possibility of referral bias from one center trial. First, the proportion of sample group was lower in group 2–4 than group 1 in present study. Second, there is a lack of knowledge about the analysis including statin therapy and differential hormonal effects based on gender. Last, our investigators suggest that prospective studies via large multi-ethnic populations and long-term follow up are required to determine the potential value of identifying the development and progression of subclinical coronary atherosclerosis and to predict the prognosis of CHD.

## Conclusions

Although there was no difference of FRS among the groups classified according to the plaque pattern on CCTA, our data suggest that more individualized therapy for reduction of cardiovascular risks could be considered in healthy subjects.
